# The slaughter of increased numbers of pregnant cows in Tanga abattoir, Tanzania: A cause for concern?

**DOI:** 10.4102/ojvr.v82i1.947

**Published:** 2015-08-12

**Authors:** Emmanuel S. Swai, Abdu A. Hayghaimo, Ayubu A. Hassan, Bartholomeo S. Mhina

**Affiliations:** 1 Ministry of Livestock Development and Fisheries (MoLDF), Dar-es-Salaam, Tanzania; 2 Tanga Municipal Livestock Office, Tanga, Tanzania

## Abstract

Information on the level of foetal wastage in slaughtered cattle in Tanzania is limited. A three-month observational study (April – June 2014) of animals slaughtered at the Tanga abattoir in Tanga region, Tanzania was carried out to determine the number of pregnant cows slaughtered. The total number of cattle slaughtered during the study period was 3643, representing a monthly kill average of 1214 and a daily kill average of 40. Over 98% of the cattle presented to the abattoir for slaughter were local breed (Tanzania shorthorn zebu) and most were above 3 years of age. Improved breeds of cattle represented only 1.3% of all slaughters. Of the cattle slaughtered, 2256 (61.9%) were female and 1387 (38.1%) were male. A total of 655 slaughtered cows were pregnant, representing a foetal wastage of 29.1%. Of the 655 recovered foetuses, 333 (50.8%) were male and 322 (49.2%) were female. Of the recovered foetuses, 25.8% were recovered in the first, 42.7% in the second and 31.6% in the third trimester. This study indicates cases of significant foetal losses, negatively impacting future replacement stock as a result of the slaughter of pregnant animals. The indiscriminate slaughter of pregnant cows suggests that existing animal welfare legislation is not sufficiently enforced and routine veterinary ante-mortem inspection of trade animals is failing to prevent the high level of foetal wastage.

## Introduction

The human population in Tanzania is projected to increase from the current 43 million to about 63.6 million by 2025 (UNPD 2008). Rapid population growth will present an important challenge to achieving food security in developing countries such as Tanzania. Statistics from the World Bank and the Food and Agriculture Organization of the United Nations (FAO) show that globally, livestock production is currently growing faster than any other agricultural sector (Robinson *et al.* 2014). This production growth is driven by the rapidly increasing demand for livestock products. The demand for meat and milk is predicted to at least double over the next two decades (Robinson & Pozzi 2011). The demand for quantity and quality of livestock products in developing countries is propelled by population growth, rising incomes, urbanisation and socio-economic factors such as human health concerns and changing socio-cultural values (i.e. change in diets) (Delgado 2005; Thornton 2010). Elasticity of demand for livestock products is four to five times higher than for cereals; if real income rises, the demand for livestock products increases faster than the demand for cereals (Tarver 1994). Livestock farming will continue to play a crucial role as a source of food, livelihood and income generation over the coming decades.

Tanzania's animal wealth at the end of 2014/2015 included 23.5 million cattle, 15.6 million goats, 7 million sheep, 2.1 million pigs, 36 million village poultry, 0.3 million donkeys and 43.2 million poultry as well as very small numbers of domestic buffalo and one-humped camels (URT 2014). More than 99% of these livestock are kept in low input–low output systems, owned and managed by resource-poor mixed and pastoral producers who operate under a traditional husbandry system, often with little or no access to good and reliable animal husbandry practices, markets and reliable veterinary services. Livestock contributes about 30% of agricultural gross domestic product (GDP), derived from an estimated 23.5 million heads of cattle, held by 1.27 million small-scale households and mostly comprised of indigenous East African shorthorn zebu. Despite its great leverage potential, the sector is seriously constrained by animal diseases, poor-quality veterinary inputs and service as a result of ineffective regulatory capacity, inadequate and low-quality feeds as a result of seasonal fluctuation, poor production or husbandry technology innovations and inadequate investments to enhance its contribution to the development of the country.

However, realisation and expansion of livestock production is constrained by other challenges, including the indiscriminate slaughter of future replacement animal stock. The trend of animal slaughter in abattoirs has shown that not only non-breeding livestock are being slaughtered for meat but also productive pregnant and lactating female animals (Adama, Shiawoya & Michael 2011; Gregory & Grandin 2007; Whitlock & Maxwell 2008). These animals are either killed for home consumption, rituals, religious festivals, ceremonies, disease control or to meet immediate financial needs (Gregory & Grandin 2007).

The wastage of conceptus through the slaughter of pregnant female animals is an unethical and uneconomic practice affecting cattle production in Tanzania. Wastage of a calf at any stage between conception and birth negatively affects herd fertility and growth. The scale and impact of slaughtering pregnant female animals is not fully recorded. There are concerns that the problem is exacerbated by the lack of proper production or market records and the failure to carry out pregnancy checks before slaughter. In order to have a clear understanding of the practices impacting the slaughter of pregnant cows, stakeholder awareness (livestock keepers and traders) and records of traded female stock for slaughter is imperative. Abattoir surveys as a tool for disease surveillance and investigation can provide essential information that can be utilised for research and disease control purposes (Cadmus & Adesokan 2009). The objective of this work was, therefore, to assess the magnitude of slaughter of pregnant cows in Tanga region, Tanzania by using an abattoir survey as a study tool.

## Materials and methods

### Study location

The study was conducted at Tanga City abattoir, located 330 km northeast of Dar-es-Salaam, the major city of Tanzania. The abattoir, constructed in 1982, provides in the daily meat requirements of the inhabitants of Tanga and neighbouring areas. Geographically, the city is located between 4°21′ and 6°14′ S and 36°11′ and 38°26′ E. Tanga City experiences tropical climate conditions, typified by hot and humid weather throughout the year. Annual rainfall is approximately 1100 mm per year, with two distinct rainy seasons: the long rain season between April and May and the short rain season between October and November. The mean annual temperature ranges from 23 °C to 33 °C on average and humidity ranges between 60% and 70% (also see Swai & Schoonman 2012).

At full operation, the abattoir has a daily maximum handling capacity of 120 heads of cattle and 150 small ruminants (sheep and goats). However, as a result of the lack of essential facilities, it presently slaughters around 40 heads of cattle daily.

### Study animals and design

The study animals were cattle brought for slaughter from all districts of Tanga region and nearby districts of Kilimanjaro, Arusha and Morogoro. Some animals were transported to the abattoir using vehicles and others were trekked in. The study design employed was an active abattoir survey, carried out from April to June 2014 (also see Swai & Schoonman 2012). The main study subject of interest in this survey was traded female cattle stock. After arrival at the abattoir, age, breed, number and origin of the animals were recorded in a purposively designed record form. The age was determined based on dentition and owner's information (Forse 1999; Turton 1999). For purposes of quality control of the data, duly filled forms were collected regularly and discussed with the meat inspector in charge. Determining the precise location of the source of each animal was not possible for various reasons, including a poor recording system and lack of reliable identification methods at farm and marketing points. No pregnancy diagnosis was conducted owing to limited facility and competency.

### Meat inspection protocol and data collection

Routine meat inspections or examinations were carried out by a para-veterinarian (the resident abattoir meat inspector with a basic animal health background) using recommended standard procedures (FAO/UNEP/WHO 1994; Gracey, Collins & Huey 1999; URT 2003). Inspection or examination procedure employed visual inspection, palpation, and incision of each visceral organ. The uteri of slaughtered cows were retrieved and the uterine horns were opened and inspected for the presence or absence of foetuses. Recovered foetuses were examined to ascertain their sex and age, estimated as described by Citek *et al.* (2011). Recovered foetuses were stratified into the categories < 3 months (first trimester), 3 to 6 months (second trimester) and > 6 months old (third trimester); these data were posted in the data collection form designed for the purpose.

### Data analysis

Data were entered, stored and analysed using Microsoft Excel and Epi-info statistical software version 6.04b (CDC 1996). Descriptive statistics such as the proportion of all slaughters, frequency of pregnant slaughtered cows and the extent of foetal wastage were generated. The percentage of foetal wastage was calculated as the total number of foetuses recovered divided by the total number of cows slaughtered.

## Ethics statement

Permission to carry out this study was granted by the Executive Director of Tanga City. The Director of Veterinary Services, Tanzania issued a research permit letter to conduct this active abattoir surveillance work in Tanga City. Verbal consent was obtained from each of the traded stock owners after explaining the purpose and importance of the study prior to data collection.

## Results

### Slaughter data

A total of 3643 cattle were slaughtered between April and June 2014, representing a monthly kill average of 1214 and a daily kill average of 40. Over 98% (*n* *=* 3596) of the cattle presented to the abattoir for slaughter were local breed (Tanzania shorthorn zebu) and above 3 years of age. Improved breeds represented only 1.3% of all slaughters. Results showed that more (61.9%, *n* = 2256) female cattle were slaughtered than male cattle (38.1%, *n* = 1387) during the period under study. The proportions of cattle (female and male) and pregnant cows slaughtered are shown in Table 1.

**TABLE 1:** Slaughtered cattle and foetal wastage in Tanga city abattoir from April to June 2014.

Month	Number of slaughtered cattle (*n*)	Male cattle slaughtered (*n*)	Male cattle slaughtered (%)	Female cattle slaughtered (*n*)	Female cattle slaughtered (%)	Foetuses recovered (*n*)	Foetal wastage (%)
April	1208	471	38.9	737	61.1	273	37.04
May	1207	468	38.7	739	61.2	202	27.3
June	1228	448	36.5	780	63.5	180	23.1
**Total**	**3643**	**1387**	**38**	**2256**	**62**	**655**	**29.1**

### Pregnant cows slaughter data

Of the 2256 cows slaughtered, 655 were pregnant, at varied gestation periods. Monthly foetal recovery rates (%) are shown in Table 1.

**FIGURE 1:**
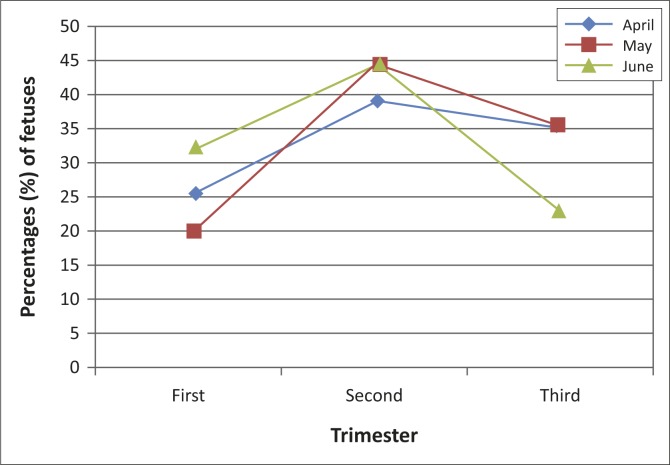
Percentage (%) of foetuses recovered by trimester and month of the survey period (*n* = 655).

The monthly foetal wastage ranged from 23.1% to 37.04%, with a mean percentage of 29.1% over the study period. This indicates that at least one out of every four cows brought to the abattoir for slaughter is likely to be pregnant. Of the total number of foetuses encountered during the survey period, 333 (50.8%) were male and 322 (49.2%) were female. Two foetuses (0.3% [2/655]) were classified as mummified. No twins or multiple foetuses were retrieved during the study. The highest recorded number of foetuses was 273 (April), of which 52.4% were female. Of the recovered foetuses, 25.8% were recovered in the first, 42.7% in the second and 31.6% in the third trimester. The highest number of wasted foetuses was recovered during the months of May and June, at the second trimester of pregnancy (Figure 1).

## Discussion

This survey revealed that 29.1% of cows slaughtered over the period April – June 2014 were pregnant. The implication of this finding is that 29.1% of the future productive herd is lost because of this practice. Reproduction losses are widely recognised as one of the important constraints to increased cattle production, and foetal wastage as a result of the slaughter of pregnant cows is definitely one of several factors contributing to low livestock production and expansion (Willingham, Shelton & Thompson 1986). Given the current livestock and marketing setting, early pregnancy diagnosis may not be possible. Lack of staff and competency in pregnancy diagnosis are possible reasons. On the other hand, there are motives for the slaughter of pregnant cows in their various stages of gestation other than lack of competency or gross ignorance. It is possible that livestock keepers or traders sell pregnant cows because phenotypically they appear heavier and consequently sell at better prices than non-pregnant ones. Limited financial resources in times of crisis, such as the dry season, may motivate indiscriminate sales of female cattle for slaughter. However, the current study was conducted during the wet season, characterised by an abundant supply of green fodder from most areas likely to be the source of slaughter stock. Furthermore, indiscriminate sales of pregnant animals could be a result of culling unwanted or non-productive cows, old or injured animals (Muhammad *et al.* 2009). In addition, some livestock keeping communities believe that foetal meat is more nutritious than meat obtained from mature cows (Muhammad, Ashiru & Abdullahi 2007) – another possible reason for the increased slaughter of pregnant cows.

In some areas and communities, particularly where this survey was conducted, evidence of pregnant dairy stock theft for slaughter is often reported. Phenotypically, pregnant cows have a better body condition score than non-pregnant cows, which may be the reason for theft preferences and slaughter of these animals.

A review of abattoir surveys conducted elsewhere showed variation in the proportion of slaughtered pregnant cows (Fayemi & Muchenje 2013). A survey conducted in Nigeria reported that 5% of the 321 448 cows slaughtered over a period of 3 years were pregnant (Cadmus & Adesokan 2010). On the other hand, results from Ghana revealed 18% (28 410/154 179) of the slaughtered cows over a period of 4 years were pregnant (Atawalna *et al.* 2013). Variations between surveys and reports could be as a result of the volume of female animals slaughtered, period and duration of the study, consumer volume, abattoir location, country-specific animal slaughter regulations and laws pertaining to slaughtering female stock.

Observations gathered from this study and elsewhere (Fayemi & Muchenje 2013) strongly suggest that the proportion of slaughtered pregnant cows is higher in those more than 3 months pregnant. Most of the foetuses recovered in this study were recovered in the second and third trimesters (74%), a finding that is consistent with reports from other studies. Wosu (1988), Ndi, Tambi and Agharih (1993) and Fayemi *et al.* (2008) found that 74%, 64.1%, and 75.7% of the foetuses recovered respectively were in the second and third trimesters.

The slaughter of pregnant cows, whether intentional or out of ignorance, has negative consequences on the reproductive potential of livestock keepers’ herds. Importantly, the high numbers of female foetuses recorded mean that an opportunity for increasing future female breeding stock is compromised. Moreover, the slaughter of pregnant cows is likely to frustrate the efforts of breeders, geneticists and nutritionists, as it poses the risk of widening the gap between the amount of meat required to provide sufficient animal protein to meet the needs of meat consumers and the amount of meat actually produced (Fayemi & Muchenje 2013).

This study was limited to visual uterus inspection, therefore we were unable to detect early embryonic foetal development (defined as the age between fertilisation and day 45) by flushing the uterus for detailed collection of embryo and eventual laboratory examination. Moreover, most of the cows presented for slaughter do not have any record reflecting their age, last calving and last service dates. This might have contributed to low early embryonic foetal recovery and detection rate in this survey.

Further studies are needed to elucidate why pregnant cows are sold and slaughtered. Such studies should focus on determining possible ways of preventing or minimising these losses. Moreover, pregnancy diagnosis should form part of standard operating procedures before any slaughter of female stock brought to the abattoir takes place. Training for extension agents, meat inspectors and farmers on simple methods of pregnancy diagnosis is recommended.

## Conclusion

This survey revealed that the equivalent of one foetus was lost for every four cows slaughtered and most of the foetuses recovered were in the second and third trimesters. The reasons for slaughtering pregnant cows ranged from sheer ignorance, cash constraint, lack of capacity to undertake pregnancy diagnosis and inability to enforce animal welfare legislation, partly as a result of the poor infrastructure available at the holding ground and slaughtering points. Policy and regulation efforts should concentrate on instituting routine veterinary checks at holding grounds and abattoirs. Livestock keepers, traders and butchers need to be regularly informed about proper animal husbandry practices, including breeding, as well as the implications of slaughtering pregnant cows.
